# “They attack the family and order”: Right-wing media about feminists and the political consequences of the women's strike in Poland

**DOI:** 10.3389/fsoc.2022.1066409

**Published:** 2023-01-11

**Authors:** Piotr Żuk, Anna Pacześniak

**Affiliations:** ^1^The Centre for Civil Rights and Democracy Research, Wrocław, Poland; ^2^Department of European Studies, University of Wroclaw, Wrocław, Poland

**Keywords:** right-wing populism, right-wing media narrative, right-wing weeklies, populist-nationalist right, content analysis, right-wing press, feminist protests

## Abstract

The goals of this article are to describe and analyze the main themes in the narratives about feminists that can be found in the populist right-wing press in Poland. Although feminists—alongside LGBT circles, refugees and the left—had been treated as a threat to the existing order before, it was the women's revolt on Polish streets in late autumn 2020 that triggered a series of reports on the women's movement in the right-wing press. In addition to analyzing the content of right-wing weeklies on women's protests, the article also shows how these protests later influenced changes in the political attitudes of young people, social activism and the attitudes of opposition parties in Poland toward cultural and moral issues. The analysis is based on the content analysis of articles published in right-wing weeklies and survey data. The authors conclude that in contemporary Poland the women's movement can contribute to overthrowing not only the rule of the populist right but also the entire conservative order resulting from the post-1989 neoliberal transformation.

## 1. Introduction: Oppressive authorities, conservative opposition, and women's protests

In October 2020, the largest street demonstrations after the 1989 systemic transformation broke out in Poland. The direct cause of the mass protests was the ruling of the Constitutional Tribunal introducing an almost complete ban on abortion (even in the case of damaged fetuses), and young girls were at the core of the demonstrations. However, these grassroots street protests soon turned not only against the rule of right-wing populists from the Law and Justice (PiS) party but also against the entire conservative system of power. The symbols of the conservative attitude were not only *dziady* [old men] (representatives of the populist government and its supporters), but also *dziadersi* (this term was used by the protesters to describe the supporters of conservative attitudes avoiding open conflict with the Church, also conservative politicians from the parliamentary opposition, as well as opponents of women's emancipation and legal abortion). From this perspective, the women's street revolt aimed at both opposing the actions of the oppressive right-wing authorities and contesting the framework of the entire official policy considered anti-woman, conservative and not fitting the standards of civic democracy. The main slogan of the protests that appeared on the first day at the head of the demonstration that passed by the Warsaw PiS's headquarters and then went toward the house of Jarosław Kaczyński, the PiS leader, was “Get the fuck out!” Although it was directed at the PiS authorities in the first place, it was addressed to the entire political system based on patriarchal, nationalist and clerical prejudices.

The aim of this article is to analyze populist right-wing narratives rejected by the women's revolt on Polish streets in late autumn 2020 and their long-term political effects. The protesting women's criticism of the conservative message, however, concerned not only statements made by the nationalist authorities and the media that supported them but also two sources of the conservative narrative. On the one hand, it was the logic, language and ideology promoted by the populist right (*dziady*). On the other hand, there were restrictions and political and moral blockades imposed by representatives of opposition parties who lost power to PiS in 2015 but were still unable to reject the conservative patterns of the current political system (*dziadersi*). The rejection of this conservative cage and the negation of these artificial restrictions in articulating political demands were the main achievement of the women's revolt. The Women's Strike negated in the public space the conservative narrative about the postulates of women's movements which was spread not only by the PiS circles but also by the right-wing weeklies that were the backbone of the ruling PiS party. The message of the populist-right-wing media about feminists, the women's movement and the protests of the Women's Strike, which is analyzed in this article, was a manifestation of a wider phenomenon which was the attack by right-wing nationalists and populists on all cultural and sexual minorities, the left understood in a broad sense and grassroots social movements criticizing right-wing authoritarianism. The final part of the article describes changes in social and political trends triggered by women's protests. Not only have young people moved politically to the left, but the liberal opposition has also become less conservative and has accepted the need to amend the abortion law.

## 2. Socio-political context of feminist protests in Poland

From the beginning of the 1989 systemic transformation, the issues of women's rights were outside the main topics of the political mainstream, and the subject of prohibiting abortion was regulated by a repressive law of 1993 allowing legal abortion in only three cases (the result of rape, when it constituted a threat to the life or health of the woman, or when there was irreversible damage to the fetus) (Żuk and Żuk, 2017). The law was referred to as a “compromise”. It was, however, a compromise not between society and the government, but between the Catholic Church and the conservative-neoliberal political scene that dominated Poland after 1989. Although since the 1990s there were women's initiatives and feminist circles that tried to change the existing situation, the slogans of social emancipation could not get through to the national forum. Abortion was practically unavailable—even in the very rare cases where the procedure could be legally performed. In practice—particularly in the provinces, rural areas and outside large urban centers—women's reproductive rights were repeatedly violated. In the atmosphere of conservative conformism and the great influence of the Catholic Church, women from small towns and villages had a problem executing the right to legal abortion and also with access to prenatal tests and reliable gynecological care (Żuk and Żuk, 2020d). There were many reasons to build a strong political emancipation movement around women's issues that would shape all politics. Hence, in the early twenty-first century, reasonable questions were asked, such as “Can the fledgling feminist movement in Poland gain ground and develop into a more influential force?” (Bystydzienski, 2001). This opportunity appeared, paradoxically, with the seizure of power by right-wing populists in 2015, the subsequent confirmation of the populist course in the 2019 parliamentary elections and a wave of new threats to women's rights, democracy and civil liberties from the right-wing authoritarian state. However, the sharpening of the political course after 2015 was a consequence of the neoliberal transformation which took place in Poland after 1989. The elements organizing the political order and the public debate on women's rights, as well as other social issues in Poland were two pillars creating ideological amalgams: (a) a combination of neoliberalism with conservatism, and (b) a relationship of right-wing populism with antifeminism, nationalism, homophobia, and dislike for any cultural differences.

### 2.1. Neoliberalism, right-wing populism, nationalism, and anti-feminism: A literature review and social science hints

What was happening in Poland was part of a larger phenomenon, that is, a right-wing populist wave, which—despite national mutations—had similar features everywhere: it combined nationalism with conservatism, neoliberalism and anti-feminism. All of this was drenched in anti-communist sauce.

Regardless of whether it occurred in Poland under Kaczyński, Hungary under Orbán, Turkey under Erdogan or Brazil under Bolsonaro, it was associated everywhere with the authoritarian patriarchalism that preached “pro-natalist policy, valorizing motherhood and calling on women to have more children ‘for the sake of the nation”' (Moghadam and Kaftan, 2019). This anti-gender and homophobic narrative of populists also spread the nationalist aversion to the European Union (EU) and the threat of “foreign influence” depicted as a threat to the family and moral order (Korolczuk and Graff, 2018; Żuk and Żuk, 2020a). This is interesting because right-wing populists in Western Europe often use the slogan of women's rights (as being threatened by the influx of Muslims) to justify anti-immigrant and Islamophobic messages (Meret and Siim, 2013). In Poland, it is the EU that is portrayed by the right as a threat to the family. Moreover, the PiS party claims that its policy supports the family and defends “Polish women” (Gwiazda, 2021a), but it is also the party that decides what is in line with women's interests and uses women's issues to perpetuate the nationalist dislike for the EU. This only confirms the opinion that the attitude of the populist right to women's rights and gender must always be analyzed in a specific cultural context and in conjunction with their views on other identities (Mudde and Kaltwasser, 2015).

While there were some local differences, generally all populist governments in Central and Eastern Europe shared heteronormative familialism, repatriarchialisation, nationalism, ethnicized demographic renewal, and anti-immigrant sentiments (Stubbs and Lendvai-Bainton, 2020). Treating the traditional family model as an expression of “free choice” limited by feminists and the left is a permanent feature of the populist right-wing narrative also in Western Europe (Mayer et al., 2014). Hence, while threats from right-wing authoritarianism to women, gender and democracy are embedded in local contexts, they require not only a national but also a transnational response (Cullen, 2021). However, what distinguishes the anti-gender messages of the populist right in Eastern Europe is their nationalistic and messianic character: the belief that Eastern Europe is the last bastion of traditional family and conservative values, offering a chance to stop moral decay coming from the West (Graff and Korolczuk, 2022). Earlier analyses have indicated that the nationalist perspective refers to gender stereotypes in Poland. According to Graff, radical nationalist mobilization is that of men in defense of traditional values and traditional social roles: “It projects the ideal feminine as the embodiment of national identity (pure, modest, and hence in need of protection), but is deeply suspicious of actual women (who now need to be watched more closely than ever, because they might be captured or themselves conspire with the enemy)” (Graff, 2009, p. 141). In this approach, spoiled and liberated women without national sympathies become a threat to the traditional order and tend to conspire with the “external enemy”. According to some researchers, these anti-gender campaigns in Poland are meant to facilitate the political mobilization of the traditional right-wing electorate, which is distinguished by greater religiosity and is afraid of too rapid cultural changes (Korolczuk, 2020). Similar elements of “moral panic” occurred in Slovenia, where anti-gender campaigns emphasized the need to save families and children (“Children are at Stake”) (Kuhar and Pajnik, 2020). In the case of the PiS party, the attitude toward gender is defined as “mostly traditional and populist”. On the other hand, in the case of the Confederation, the second right-wing populist parliamentary party (which is a cluster of nationalists, traditionalists and conservative neoliberals), the attitude toward gender is described as “ultra-traditional and populist” (Gwiazda, 2021b).

While the above-described relations between right-wing populism and antifeminism are clear and do not raise any doubts, it is necessary to explain the relations between neoliberalism and conservatism under the conditions of Eastern Europe. In the early 1990s, Szacki (1994) emphasized that Polish (Czech, Hungarian) liberalism was only economic, not moral or political. Moreover, the liberalism of the 1990s was primarily a scourge on the left in post-communist countries and part of the tradition of “liberal-conservative” right-wing politics from the very beginning. Anti-communist liberalism was more attached to market fetishization and fighting “leftist” traditions than to defending individual autonomy or liberal freedoms. Additionally, the liberals made it clear that, by building the “kingdom of the market”, they would willingly take advantage of the state's coercion. These authoritarian inclinations of the liberals in the post-communist world were illustrated by the words of Stefan Kisielewski, one of the ancestors of Polish liberalism: “Take them by their gobs and introduce liberalism” (cf. Szacki, 1994, p. 188). These words expressed intentions consistent with beliefs of the sympathizers of neoliberalism, who agreed with the opinion expressed by Friedrich von Hayek in the context of Pinochet's policy in Chile: “it is possible for a dictator to govern in a liberal way. And it is also possible for a democracy to govern with a total lack of liberalism. Personally, I prefer a liberal dictator to a democratic government lacking liberalism” (Szacki, 1994, p. 191). This right-wing and anti-communist trait of East European liberalism makes it possible to better understand why right-wing populists can be neoliberal despite being described as “illiberal” [they restrict media freedom, attack the independence of the judiciary, fight independent civil society and use anti-gender rhetoric (Zvada, 2022)]. The illiberal is not in conflict with the neoliberal. It is seemingly a kind of paradox because, on the one hand, right-wing populists use the social rhetoric of “the defense of ordinary people”, trying to gain political support, but, on the other, after gaining power, they accept the neoliberal logic and become a “new elite” themselves. This was clearly visible in Poland.

Sympathies for PiS and right-wing populism were a consequence of confusion and an expression of frustration caused by the effects of neoliberal politics. This was particularly true of working-class groups and lower classes, for whom the nationalist-populist wave was a reaction to a sense of economic degradation (Żuk and Żuk, 2018a). The relationship between the neoliberal policy and its effects was visible in many places of social reality. For example, the so-called homophobic “LGBT-free zones”, were created in 2019 and 2020 by some local PiS authorities primarily in the regions that suffered the most during the neoliberal transformation in the 1990s and in which “special economic zones” were first established in the 1990s, becoming a symbol of the neoliberal economy (Żuk et al., 2021).

In Poland, the PiS government tried to organize “joint sales” to gain support for its politics: on the one hand, it limited civil rights and built the foundations of an authoritarian state; on the other, it promised social assistance for families in the form of a “500 plus” child allowance program (Pacześniak, 2018). Although this program did not change the general neoliberal logic of state economics, it gave the impression of a substitute for social assistance for large families (Shields, 2019). Although the nationalist populism that spread across Eastern Europe 30 years after the fall of the Berlin Wall was a cultural and political reaction to the neoliberal transformation carried out in the region (Żuk and Toporowski, 2020), the populists in power remained neoliberal. The only change was the belief in the possibility of gathering “national capital” that the populists wanted to create as their own base. East European populists “have sought to develop a national capitalist class, while balancing reliance on Western capital through closer economic relations with authoritarian countries” (Orenstein and Bugarič, 2022). The mixture of authoritarianism, nationalism and neoliberal domination in an economy driven by a belief in better because “national capital” is actually a trademark of right-wing populists in Eastern Europe (Fabry, 2019; Shields, 2021) and other countries across the world (Akçay, 2021; Ramos, 2021).

## 3. PiS's election victory in 2015, a populist turn and the revival of the fight for “lost cases”

After PiS came to power in 2015, the Catholic Church expected a complete ban on abortion to be introduced into Polish legislation. The political relationship of the Church with the right was in force from the beginning of the 1990s and was not only limited to the issue of abortion but defined the general framework for public debate. The seizure of power by PiS strengthened this alliance between the throne and the altar (Żuk and Żuk, 2019). The common language of dislike for gender, feminism and LGBT rights (Żuk and Żuk, 2020c) used by the right and the Church was supposed to consolidate the existing conservative order and also to implement a cultural counter-revolution, after which it would be impossible to reverse many of the legal and political changes introduced (Koczanowicz, 2016). The tools of the cultural and ideological counter-revolution were to include the current politics and also the educational system and school curricula subordinated to the nationalist narrative, media coverage, and the politics of memory (Stańczyk, 2018).

### 3.1. PiS's anti-woman populism and a feminist response

As PiS took over further state institutions (public media, the civil service, prosecutors, and special services) (Markowski, 2016), the pressure to control the cultural and moral sphere was also growing. In early 2016, the PiS government signaled that it was able to support a complete ban on abortion. However, the PiS leaders wanted this decision to give the impression of a government response to grassroots “social demands”. Signatures in support of prison sentences for both women and gynecologists collected by the clerical organization Ordo Iuris were to be the pretext for imposing a nearly total ban on abortion. Half a million signatures on this bill collected by Ordo Iuris allowed the ruling party to enter a legislative process that would completely criminalize abortion (Kubisa, 2017). This caused public anger. On 3 October 2016, the streets of Polish cities were flooded with thousands of protesting women dressed in black and using the slogans “These are my ovaries,” “Freedom, Equality, Right to Abortion,” “Your Parliament, Our Bodies” (Narkowicz, 2016). The scale of the protest surprised the authorities and ultimately caused PiS to withdraw from this legislative bill. However, something else happened. As Korolczuk (2017) writes, “[t]o many, the All-Poland Women's Strike (Ogólnopolski Strajk Kobiet) became a revolutionary moment when fear and anger were transformed into feelings of solidarity and empowerment, a moment of personal and collective transformation”. Others emphasized that a new movement had been born and that this new wave of feminism in Poland was fueled by a new generation for whom Black Protests turning into a formative experience for many of the previously non-active participants could change the Polish reality (Hall, 2019).

It took 4 years for the social anger sown in 2016 to bear fruit, show its mass character and formulate a more radical demand for socio-political change. In the meantime, PiS won the parliamentary elections for the second time in 2019. By repeatedly breaking the constitution and limiting civil rights, it consolidated its power, confirming its anti-liberal and anti-European stance (Markowski, 2020). Therefore, after the next victorious parliamentary elections in the autumn of 2019, it continued its ideological offensive.

On 22 October 2020, almost exactly 4 years after the birth of the All-Poland Women's Strike, the Constitutional Tribunal, which was deprived of independence, and completely controlled by and subordinated to the power of PiS (Sadurski, 2019), found the permissibility of abortion unconstitutional “when prenatal tests or other medical conditions indicate a high probability of severe and irreversible fetal impairment or an incurable disease that threatens its life” (Trybunał Konstytucyjny, 2020).

It was a signal for enormous social mobilization on the streets of Polish cities. The protesters' criticism was directed not only at the PiS authorities but also at all those forces that represented the fossilized and conservative political system. Referring to conservative commentators on the street rebellion, the leaders of the All-Poland Women's Strike wrote as follows:

“It is time to finally enter the twenty-first century, dear commentators, who hug traditional politics that evoke laughter and embarrassment among young people under the conditions of a revolution. We have had enough of the ossified nineteenth-century hierarchy and the grace of the rulers, enough of the twentieth-century political marketing projects and gently smiling gentlemen who proclaim themselves leaders of the revolution when police gas is sprayed at us. We can decide for ourselves. We can take care of ourselves during protests; we will also take care of ourselves after them. It is time to clean out old cupboards and give power to people, including young people who do not want to live in the country of conservative old men” (Lempart and Suchanow, 2020).

The events that took place in Poland in the autumn of 2020 not only indicated a social awakening and the activation of wide-ranging groups in the fight for political emancipation. It was also about a symbolic change in the narrative and the negation of the existing rules of the socio-political game. As Agnieszka Graff aptly wrote:

“Something has broken, something has spilled out. A certain compromise has ended in Poland. However, it is not about an ‘abortion compromise' here, because nothing like that existed, but about a much broader systemic compromise. The Great Compromise between the state and the Church, the one on which the order of the Third Polish Republic and the identity of Poland after 1989 were based” (Graff, 2020).

## 4. Data and methods of analysis

The empirical material used in this article comes from several sources. Firstly, to show the main elements of the right-wing narrative about feminism, the message of three right-wing weeklies, which are PiS's media support and also its ideological outlet, has been analyzed. The analysis of the content of *Do Rzeczy* [*To the Point*], *Gazeta Polska* [*Polish Newspaper*], and *Sieci* [*Networks*] has made it possible to describe the most frequently repeated themes of the right-wing ideology regarding feminism in Poland. The selected titles of the right-wing weeklies and their online portals are linked not only ideologically and politically with the PiS party but are also generously supported financially by the PiS government through state-owned companies (Polityka, 2019). In this sense, the message of these selected weeklies should be treated not only as a narrative of the right-wing populist press but also as the PiS party's propaganda.

The selection of material primarily included publications from 2016 to 2020, that is, from the period when PiS was in power (although earlier publications of these newspapers were also used). The selection of articles was simple: materials containing the terms “feminism,” “feminists,” and “All-Poland Women's Strike” were searched for using search engines on the websites of the aforementioned weeklies. Moreover, to show the impact of the Women's Strike on the change of political trends in Poland, the results of a national survey on changes in attitudes toward the right to abortion, participation of young people in protests, as well as changes in political sympathies in the young generation in Poland (primarily among women) were used.

The analysis of the collected material is inspired by the influence of Wodak (1997) and the belief that the use of language is always a form of social practice which, on the one hand, shapes the social reality and, on the other hand, is a manifestation of the existing social structure and dominant ideologies. However, this is not a typical discourse analysis. The analyzed types of conservative narratives are meant to illustrate the framework of a limited public debate denied by the women's revolt. The presented narrative of the right-wing media about the women's movement also illustrates the cultural and political world in which the populist-nationalist right in Poland has entrenched itself.

## 5. Results and discussion

### 5.1. “The secret plan of the ideological revolution”: Right-wing media narrative about feminists and the women's movement

The analysis of the way feminists and the women's movement are portrayed allows us to agree with the thesis that right-wing populists, just like authoritarian and totalitarian regimes, must constantly fight the omnipresent enemy (Arendt, 1973). The women's movement played the role of another public enemy and in the right-wing message, it became an element of a secret plan to carry out an ideological revolution. As Tekieli warned in *Gazeta Polska*, the “silent revolution” that threatened traditional values had its own cunning plan:

“The rainbow plague is part of a much broader revolutionary plan that is a network and decentralized phenomenon. The revolution is to go quietly. It involves the combined forces of communities related to LGBT ideology, that is, gender ideology, radical feminism, neo-Malthusianism, extreme environmentalism, esoteric New Age and the latest post-Marxist left” (Tekieli, 2019).

PiS leaders and their media supporters more or less directly used Carl Schmitt's legacy to manage social conflict in accordance with the following principle: find an enemy, describe them and attack them. All power is to be exercised in the name of the “sovereign” and any ideologies that threaten national sovereignty are to be rejected (Schmitt, 1985). Just as the attacks on refugees and Muslims helped PiS gain power in 2015 (Żuk and Żuk, 2018c) and then strengthened it, the same goals were served by the subsequent attacks by public television (Żuk, 2020) and the PiS-supporting press on the LGBT community (Żuk and Żuk, 2020b). Also, anti-Semitic accents were to strengthen the “national identity” and the “national politics of memory” in the media coverage. For similar reasons, feminists and the women's movement were well-suited to be another “surrogate enemy” which threatened tradition, religion, the Church, family, and Polish morality. Both the public media appropriated by those in power and the quoted weekly newspapers connected with PiS never concealed their dislike for feminism and based their attitude on close relations with the Church, treating the gender perspective as an element of a foreign ideology and a mixture of conservative and populist beliefs (Gwiazda, 2020).

The narrative of the right-wing weeklies illustrates not only the views of the PiS milieu (called *dziady* by feminists) on the issues of the women's movement but is also a mirror of the general cultural and political beliefs of the representatives of the populist right.

Janicki, a political journalist, has very aptly described the category of *dziady*. In his opinion:

“*Dziady* are deeply conservative men, supporters of ‘natural' patriarchalism, a traditional family with the division of gender roles, emphasizing their religiosity and ties with the Catholic Church. … They support a complete ban on abortion, are against same-sex relationships and often also against IVF. They sometimes pretend to be modern, but prefer a religious, ideological and ethnic community to ‘human rights.' And also to civil liberties, and thus—the constitution, the tripartite division of powers, the independence of institutions and similar ‘abstractions.' Politically, they are mostly located in PiS and its vicinity” (Janicki, 2020, p. 20).

On the cultural and political level, “*dziad*y are simple, unashamed opponents of cultural and moral progression, who do not value liberal democracy and rely on a familiar, clan community of brothers-in-law” (Janicki, 2020). In the individual dimension, they are model PiS voters, who accept all cultural and political dogmas on which the power of the right-wing populist state is based.

### 5.2. Nasty women are fighting for grants, not for women's rights

The language of *dziady* flourished in the right-wing press for many years and was not only limited to conservative and right-wing men. In 2013, even before the PiS party took power in the state, a *Gazeta Polska* journalist accused feminists of “dehumanizing” women, and reduced their motivations to “fighting for grants”:

“The feminist slogan ‘My belly belongs to me' is intended to encourage abortion and dehumanize women, who are already despised by gender ideologues. I have not noticed that feminists are fighting for the dignity of women, for the dignity of the mother, for the dignity of the family, they are fighting for their positions and grants, generously paid by the ‘elites' of political correctness. … The worst thing, however, is that thanks to the activities of these disgusted and often ugly jades, more and more women lose their self-esteem, accepting the lifestyle promoted by the media guys and their unscrupulous female helpers” (Grzybowska, 2013).

Putting feminists in line with the “elites of political correctness”, the author of the article used a typical populist narrative which referred to the division into “ordinary people” and “elites” (Müller, 2017). In this case, feminists are equated with “elites” who are the opposition of “the dignity of the mother” and “the interests of ordinary women”. From this perspective, feminists are only interested in careers and obtaining grants, and are ready to attack religion, the Church and traditional values in the name of these goals.

### 5.3. Feminists worse than communist police: They attack government, religion, and the church

After the PiS won power, feminists were identified as an “anti-government” factor. In April 2016, the article entitled “Feminists worse than communist police,” which appeared in the weekly *Sieci*, warned:

“Polish feminists have entered a new phase of struggle. They will be an excellent destabilizing factor, helpful in countering government actions. How far will they go? All initiatives so far have been a faithful copy of Western solutions. Does the entrance to churches herald the duplication of successive barbaric happenings that no one has yet dared to do?” (Nykiel, 2016)

Later in this article, the author warned against “blasphemous happenings” organized by feminists in various countries of Western Europe. In this narrative, the West is a synonym for corruption and demoralization against which Poland should defend itself. According to the author, “leftist activists know no boundaries. They will push them until they meet resistance”. The right-wing columnist wrote in 2016 that “the pathological game of feminists is just getting started. With a coat hanger in hand and the slogan ‘cursed wombs', they will demand legalization of abortion, repeating mindlessly that they have full rights to their own bodies”.

The title comparison of feminists to communist police units used to break up demonstrations was, firstly, supposed to show the readers that the “symbolic violence” practiced by feminists was worse than the physical violence of communist police units. Secondly, it was aimed at emphasizing the continuity between communism and feminism. This narrative cliché is often used in the right-wing media in Poland.

### 5.4. The feminist movement as a new incarnation of Marxism and a repetition of communism

Right-wing journalists and politicians regularly resort to acts of persuasive inference against their opponents. Persuasive inference consists of conscious exposure to similarities between two phenomena and simultaneous concealment of all differences between them. This is done to discredit the subject of the comparison by identifying it with a phenomenon that is treated as discredited or lost at the level of common knowledge (Polkowska, 2015, p. 131). The accusation of leftism and connections with communism is also raised against LGBT communities, feminists and environmental activists.

During the ongoing street protests, the article entitled “The Bolshevik liberation of women” in the weekly *Do Rzeczy* announced that “the demands put forward by activists of the so-called Women's Strike often refer directly to the slogans proclaimed by the Bolsheviks over a hundred years ago and implemented during the revolution in Russia” (Szumiło, 2020). According to the author of the article, the Bolshevik sexual revolution inspired feminists and “sex specialists” in Western European countries, and now the same wave is trying to break into Poland. The favorite slogan of right-wing propaganda in Poland is—next to the word “leftism”—the term “neo-Marxism”. Right-wingers use this concept in relation to all progressive cultural changes. They associate them quite freely with the name of Herbert Marcuse and the Frankfurt School. In practice, however, the slogan “neo-Marxism” refers to all ideas that are contrary to the traditional teaching of the Catholic Church and to the assumptions of conservative nationalism. As a right-wing journalist warns:

“Neo-Marxism, which refers, *inter alia*, to the idea of ‘liberating' the woman, or breaking up the family, and ‘bourgeois morality', has practically won in almost the entire Western world. Poland is still resisting these influences, which the left-wing feminists regret and are trying to fully introduce here the gains of the Bolshevik sexual revolution from before a hundred years ago” (Szumiło, 2020).

In the weekly *Do Rzeczy*, Warzecha has noticed other similarities between feminism and communism. According to him, although women's rights are respected, “feminist fighters argue that the fate of women is still terrible and requires extraordinary measures. As in the theory of communism—as the revolution progresses, the class struggle becomes more intense” (Warzecha, 2018). According to the author, feminists are an example of the followers of “the most paranoid conspiracy theory”, the manifestation of which is to be seen everywhere as “patriarchal conspiracy”. As Warzecha (2018) claims, feminist paranoia makes it impossible, for example, to recognize that for “natural reasons” “women are less interested in politics…, and prefer to devote themselves to the home”. What is supposed to connect feminism with communism is the will to implement one's own ideological projects with the help of the social engineering of the state.

On the other hand, in *Gazeta Polska*, the relationship between feminists and communism is understood literally through family ties. *Gazeta Polska*, which seeks direct ties with the functionaries of the former communist system in various circles, has already used these practices. This was the case, for example, with the media campaign against judges who protested against the violation of the rule of law and the independence of courts by the PiS authorities. This was also the case with representatives of local authorities who sympathized with other political options than PiS. This was also practiced in the case of leaders of the feminist movement in Poland, who were said to have connections with people of the former communist regime. For example, it was revealed that Graff, one of the most famous feminist academics, came from the family of a military prosecutor from the Stalinist period. Katarzyna Bratkowska, involved in gender studies, was reproached for her parents' journalistic career during the communist period (Marosz, 2014). This type of personal attack and an attempt to create a conspiracy theory linking the former communist system with activists of the modern women's movement is a common phenomenon in the circles of the anti-communist right in Poland. If contemporary feminism in Poland is said to be a hybrid of communism and neo-Marxism, right-wing propaganda can trace terrorist activities in it as well.

### 5.5. From feminism to terrorism: Feminists attack public order and the police

As right-wing populists try to discredit the activities of the environmental movement by calling them “eco-terrorism”, the feminist movement can also be subject to similar accusations. The hot autumn of 2020 on Polish streets not only angered the right-wing government but also scared its media. *Gazeta Polska* warned that this could be the beginning of leftist terrorism in Poland. The leaders of the Women's Strike were compared to the activists and leaders of the Red Army Faction (RAF)—Ulrike Meinhof and Gudrun Ensslin. As Witold Gadowski wrote:

“The decision of the Constitutional Tribunal is only a pretext here to trigger a nationwide wave of terror, for which left-wing activists have been preparing for a long time. When I watch the shaky speech and facial expressions of the leaders of the so-called Women's Strike, I can irresistibly see the faces of Gudrun Ensslin, Andreas Baader, Ulrike Meinhof and Birgitte Mohnhaupt. The same hysteria, uncontrolled outbursts of aggression, curses and absurdity of postulates. These associations, however, evoke shudders, madness and ferocity, because they are a spark that, when it falls on gunpowder, can trigger an uncontrolled explosion” (Gadowski, 2020).

The weekly *Sieci* also unleashed hysteria over alleged feminists' attacks on the police and the radicalization of the street revolt which, according to right-wing journalists, threatened the public order (Pyza and Wikło, 2020). In fact, it was the police who used violence and repression to stifle street protests.

Scaring the public about the violence allegedly used by feminists was to discredit their activities and at the same time was a call to criminalize their activities. The practice of criminalizing social movements, seen previously in Latin American countries (Doran, 2017), makes civic activity a synonym of criminal behavior requiring legal sanctions from the state repression apparatus.

### 5.6. Sushi-feminism: Snobbery of celebrities, elites, and middle classes

Right-wing populists refer to the opposition between ordinary people (“ordinary,” “honest,” “our” people) and elites (corrupt, cosmopolitan, arrogant). Populism also suggests that politics should be an expression of the *volonté générale* (general will) of the people (Mudde and Kaltwasser, 2017). In this context, the Polish right clearly states that feminists have nothing to do with “ordinary women” and are merely an ideological product promoted by celebrities at the level of mass culture. Wildstein wrote in *Gazeta Polska* as follows:

“Both environments, of feminists and celebrities in a broad sense, have many common features. They are characterized by the so-called disdain for reaction, disgust at ‘conservative views' and ‘religious women'. And also by the fetishization of sexual freedom and flaunting it…, having dressed it in the garments of ‘progress' and ‘independence'. Both groups adhere to a specifically understood ideal of ‘individualism', simply trivially reducing it to contestation of family, national and religious obligations” (Wildstein, 2011).

The conservative right criticizes sexual freedom in its traditional way and also contrasts the apparent “individualism” of feminists with family, national or religious obligations. However, this narrative also contains an element of “class” thinking that is worth a closer look. According to the *Gazeta Polska* journalist:

“… feminism begins to represent the interests of a specific, already emancipated, wealthy and influential group. In this way, it turns into sushi-feminism, that is, into a coarse ideology, through which all currently top media stars can ennoble themselves and give themselves the status of authorities” (Wildstein, 2011).

This thread of celebrity feminists who have nothing to do with the fate of ordinary women was repeated many times in right-wing weeklies. In 2017, Kołodziejski even defended the thesis that the opponents of feminists were “ordinary women” who led a traditional family life. In this approach, feminists do not fight the patriarchal political system, but, as the author said:

“… their natural enemy is ordinary women who—instead of getting excited about oral sex scenes with a pope—run after work to pick up their child from school and… make sense of it. Militant feminists are unable to understand it, reject femininity and motherhood and hate families” (Kołodziejski, 2017).

Generally, these mythical “ordinary women” come from the popular classes as opposed to big-city feminists.

Interestingly, this class aspect of contemporary feminism has also been highlighted by Slavoj Zizek, unrelated with right-wing populists, who has noted that:

“… feminist struggle can be articulated into a chain with a progressive struggle for emancipation, or it can (and it certainly does) function as an ideological tool of the upper-middle classes to assert their superiority over the ‘patriarchal and intolerant' lower classes” (Zizek, 2005).

Is feminism really limited to middle-class circles and part of its ideological and cultural label? Does this prospect condemn women of the working class to a conservative and right-wing populist vision of the world? How can this class-cultural dilemma be overcome?

It seems that the ideological dichotomy between “ordinary women of traditional values” and “upper-middle class members oriented on market success and using the feminist label for marketing purposes” created by the right-wing narrative can be challenged in one way. Instead of answering the question of which is better: redistribution (related to class politics) or recognition (associated with identity politics movements) (Fraser and Honneth, 2003), both redistribution and recognition should be demanded. In this sense, one cannot separate the postulates of greater cultural, moral and political freedom from equality issues and the postulates of greater egalitarianism. According to this approach, the feminist movement should express the demands of both the “cultural left” and the “economic left” to fill the artificial division between them. In political practice, this would question the division promoted by the populist right into “middle-class liberals waving the banner of political correctness” and “patriotic popular classes”. Similar hints were made by Nancy Fraser regarding the situation in the United States after Trump's victory in 2016. There, conservatives successfully mobilized the lower classes against “progressive neoliberalism” understood as “an alliance of ‘new social movements' (including feminism), on the one side, and the high-end ‘symbolic' and service-based business sectors (Wall Street, Silicon Valley, and Hollywood), on the other” (Fraser, 2016). From this perspective, right-wing populism can be counterbalanced by a “new feminism” in alliance with a “new left”, combining the postulates of redistribution and recognition.

### 5.7. The covers of right-wing weeklies warn: “From red Moscow to rainbow Brussels”

Contrary to what the populist right claims, the phenomenon of feminism is not something marginal for it. This is evidenced by the fact that feminists often become the “subject on the covers” of the right-wing weeklies. The slogans and the message used on the covers harmonize with the content of the articles contained therein and are part of the message of the conservative anti-feminist ideology. The way in which feminists are presented on the covers is to evoke an aversion to them, ridicule them and perpetuate conservative stereotypes.

**Figure 1:**
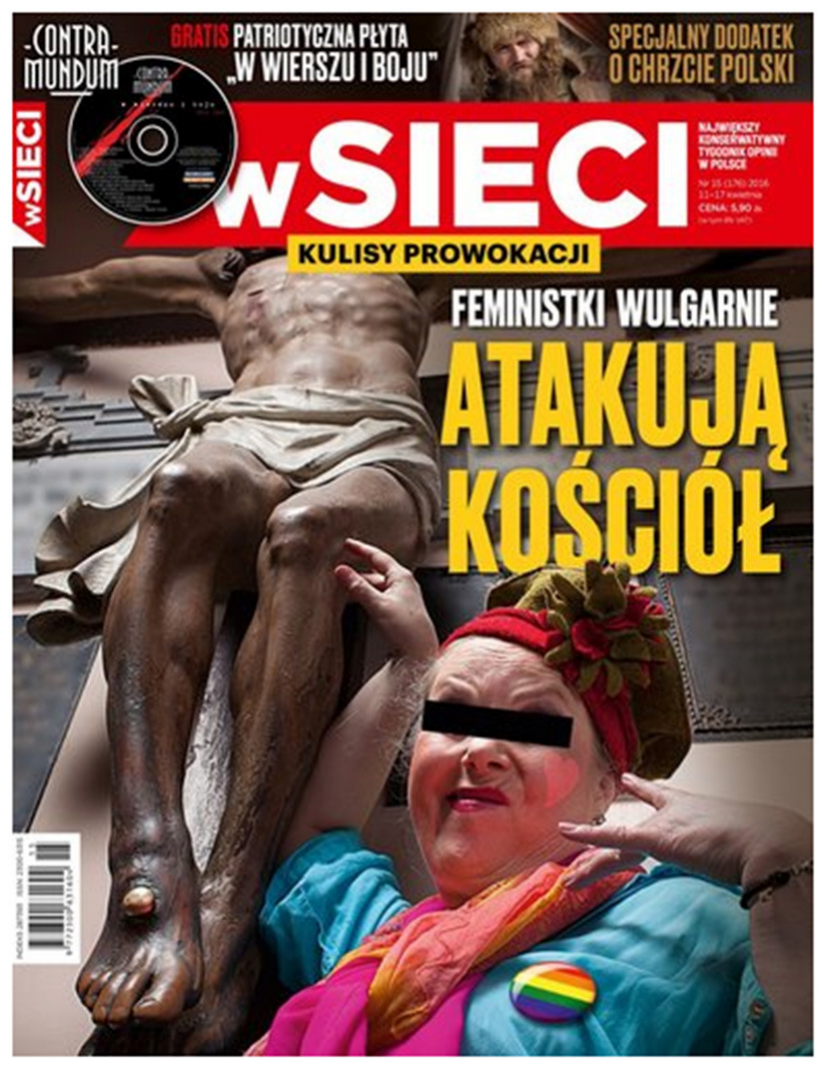
The cover of *Sieci*, no. 15/2016.

The 2016 cover of *W Sieci* [*In the Network*, now *Sieci*] (Figure 1) portrays the figure of Jesus on the cross profaned by a woman with a belt on her eyes (this is how people suspected of a crime are anonymized and, at the same time, stigmatized). A badge with a rainbow flag attached to the woman's clothing is to symbolize “gender” and the feminist movement. The cover title leaves no doubt: “Feminists vulgarly attack the Church”.

**Figure 2:**
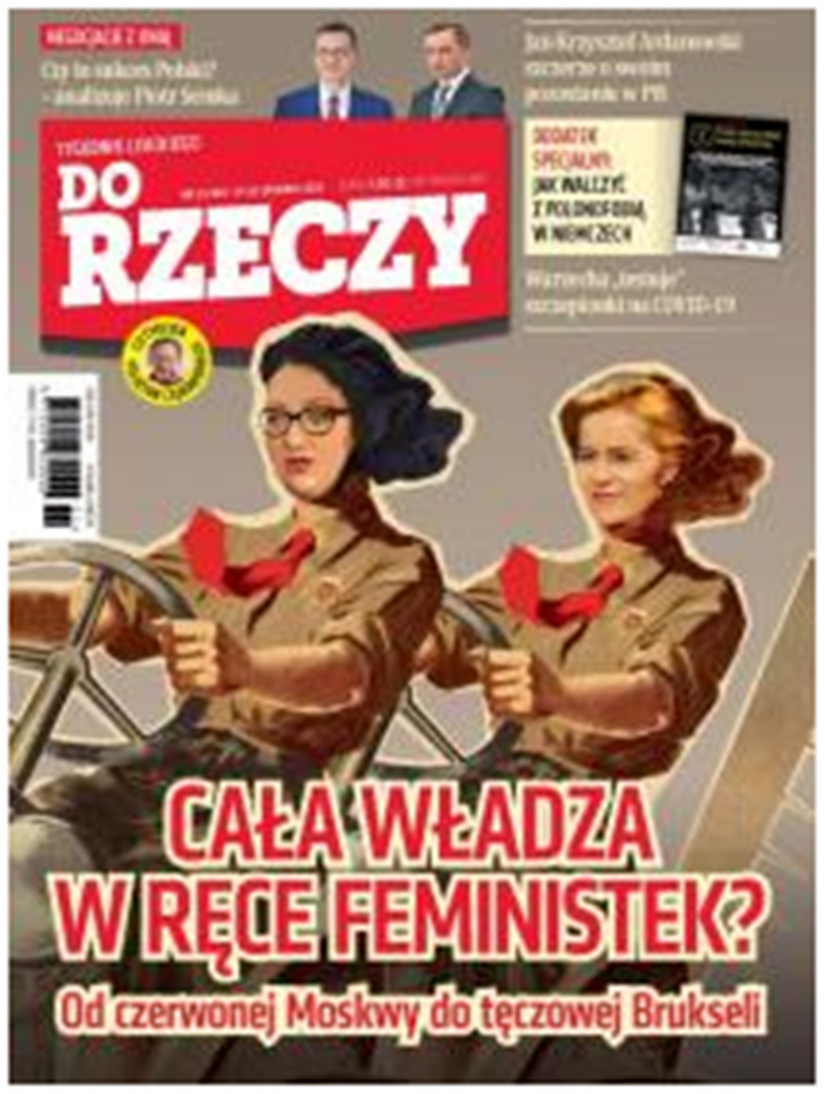
The cover of *Do Rzeczy*, no. 51/2020 of December 2020.

On the cover of *Do Rzeczy* from December 2020 (Figure 2), we can see a drawing of the leader of the Women's Strike (Marta Lempart in the foreground) and women styled as activists of the Polish Youth Union (*Zwiazek Młodziezy Polskiej*—ZMP, a communist youth organization operating in Poland during the Stalinist period, modeled on the Soviet Komsomol). In the drawing, feminists are dressed in ZMP uniforms and red ties, with ZMP symbols pinned to their lapels. The cover title “All Power to Feminists” refers to the communist slogan “All Power to Councils.” The subtitle “From red Moscow to rainbow Brussels” refers to the right-wing anti-colonial discourse. This slogan is intended to clearly show the similarity between the subordination of Poland to the Soviet Union during the communist period and the subordination of Polish interests and tradition to the political correctness imposed by the EU today.

**Figure 3:**
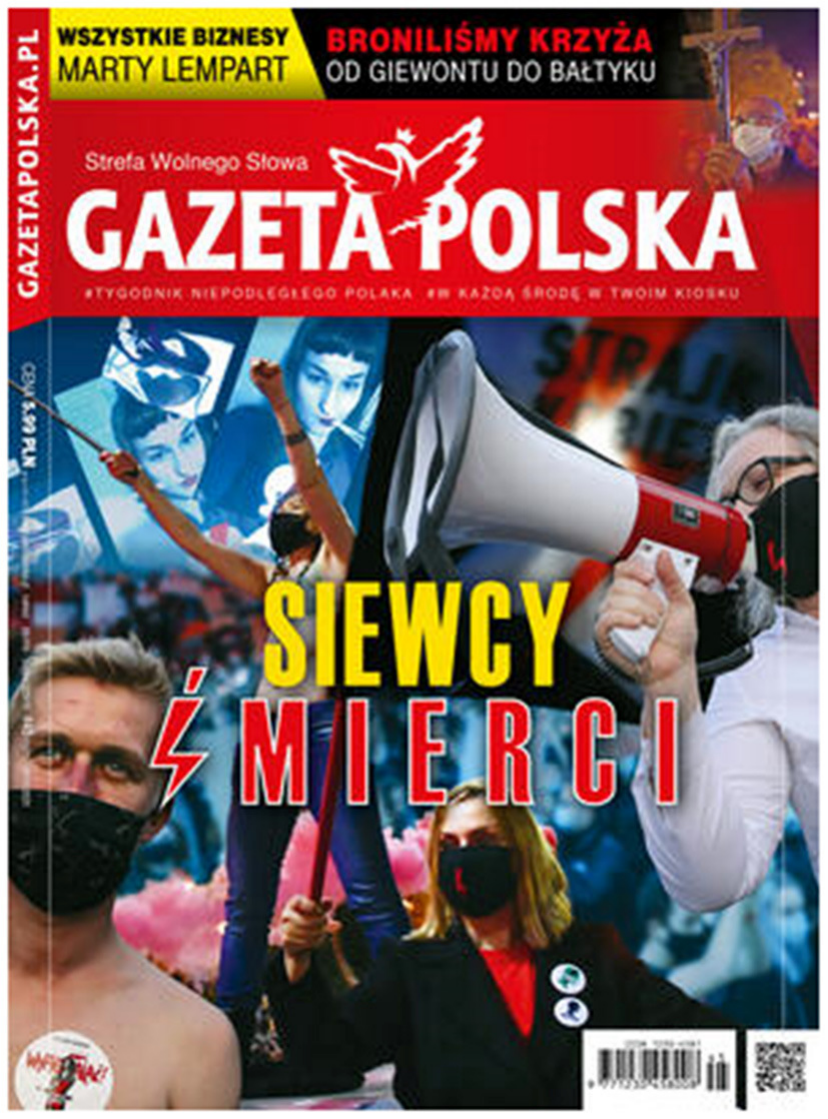
The cover of *Gazeta Polska*, no. 45 of 4 November 2020.

In turn, the cover of *Gazeta Polska* from November 2020 (Figure 3) shows a collage of photos from the women's demonstrations (one of them shows Marta Lempart, the leader of the Women's Strike, with a megaphone in her hand). The slogan under these photos is “Death-sowers” (the word “death” is made from the red lightning—the symbol of the Women's Strike) has a clear meaning in the context of the ongoing pandemic: demonstrating women spread the virus and sow death.

**Figure 4:**
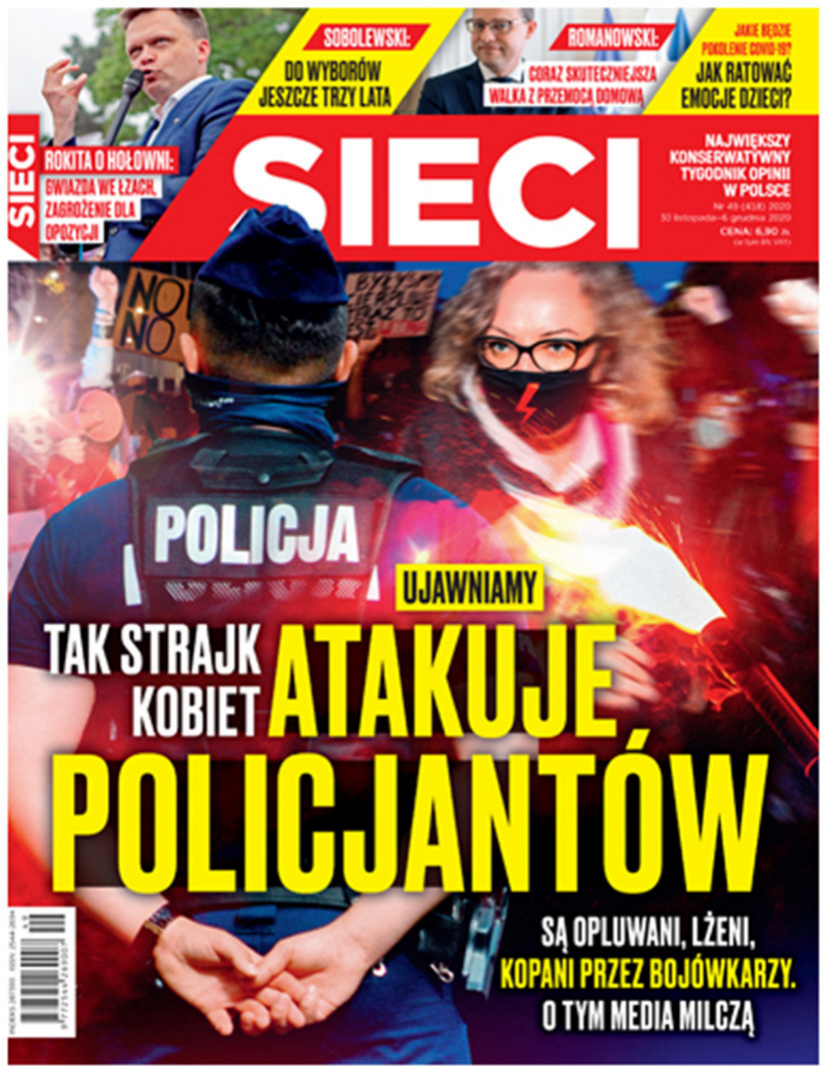
The cover of *Sieci*, no. 49/2020 of 30 November 2020.

The anarchist and rebellious face of the Women's Strike is emphasized on the cover of *Sieci* from 30 November 2020 (Figure 4). In the background we see the demonstrations taking place at that time, and in the foreground, there is Marta Lempart again. Her face is covered with a black mask with the red lightning. On this cover, Lempart stands angrily and provocatively in front of a policeman. The cover title reads: “Yes, the Women's Strike Attacks Police Officers.” A smaller subtitle has been added: “They are spat on, insulted, kicked by militants.” The cover design reinforces the right-wing populist opinion that the Women's Strike and the people demonstrating in the streets are in fact a “brawlers' strike” and a hooligan revolt used to weaken the PiS's power. The right-wing media tried to impose on Marta Lempart the image of a rude, unbalanced, law-breaking, arrogant person, from whom all reasonable people should cut themselves off and whom they should also condemn.

## 6. *Dziadersi* in stride: Do not break the compromise, do not be too radical, change so that nothing changes

Criticism of the political order in Poland from a feminist perspective covers not only *dziady* (identified with the ruling right associated with PiS) but also *dziadersi*.

“A *dziaders* is a man less conservative than a *dziad*, pro-European, preserving democratic standards, defending the constitution and the rule of law, but at the same stuck, even unconsciously, in the old patriarchal mentality. He considers himself a moderate centrist, a representative of the mainstream, but, according to critics of this attitude, he does not notice his own ideological entanglements and the fact that he is stuck in social stereotypes, that he perceives today's reality in terms of the 1980s and 1990s, when he first fought for a new Poland, and then strengthened it” (Janicki, 2020).

Although a *dziaders* is not a clericalist and it is difficult to accuse him of a completely submissive attitude toward the Catholic Church, he is:

“… a bit ‘churchy' because he was brought up that way and that is what remained in him. He is liberal, but in a conservative-liberal way, so he has his limits, he is sometimes focused on the economy, entrepreneurship, budget and deficit. Everything in him is in turmoil against ‘giving money to people'. He is deeply distrustful of the revolution. … He calls for peace and pragmatism, and is unclear and reluctant to speak about sensitive, worldview matters. He grimaces at radicalism and inappropriate vocabulary” (Janicki, 2020).

This description fits well with the atmosphere prevailing in the Civic Platform, the main opposition party in Poland, and the agrarian Polish People's Party which was a junior coalition party of the Civic Platform from 2007 to 2015, and co-ruled with the Alliance Democratic Left from 1993 to 1997. It also applies to some representatives of the parliamentary left, particularly those coming from the post-communist Alliance Democratic Left.

A good illustration of the *dziaders'* attitude can be the following statement by Grzegorz Schetyna, one of the leaders of the liberal opposition, who said during the women's street protests:

“I do not want abortion on demand. I believe that you need to draw conclusions from what happened, that we need to calmly talk about how to return to the abortion compromise and modernize it in some way, give it a new life after nearly 28 years of being in force because it saved us from the civil war over abortion. … If someone says: either abortion on demand, or we have nothing to talk about, all or nothing, this means nothing, this means that they do not know what politics is, they do not know what a compromise is” (Wiśniewska, 2020).

Bogdan Zdrojewski, a former minister of culture in the Civic Platform government and also one of the leaders of the Civic Platform, appealed for restraint when the police used violence against women protesting in the streets and the entire Women's Strike movement. He also called for not succumbing to the “extreme left” with which he identified the women's protests. According to him, the opposition “should not succumb to the expectations of the extreme left, which do not create any limitations, such as those resulting from the progress of medical knowledge. We need to be cautious, adequate and restrained here” (wPolityce.pl, 2020). Identifying feminists with the extreme left and the threat of revolution, as well as the threat to “social peace”, was the common denominator of the ruling populist right and the liberal-conservative opposition.

It is hardly surprising that, in these circumstances, the women's circles clearly emphasized that their protests not only concerned the rule of *dziady* but also the framework of politics and public debate created by the opposition *dziadersi*. In her appeal to *dziadersi*, Kaja Puto wrote:

“Dear *dziadersi*, women are taking to the streets to demand their full civil and political rights and warn that they will demand this from any politician who raises their hand at these rights. Over the past 30 years, when you have been saying the same conservative nonsense over and over again, Polish women have changed a lot. The time of free laundresses, cooks, psychologists, assistants and secretaries who can never count on your reciprocity is over… The time of your exclusivity in the public debate is over. Girls who are taking to the streets—and those aged 15 to 45 dominate among them—will take care of this” (Puto, 2020).

Protesting women and feminist activists emphasized that when using the term “*dziaders*”, they do not mean the age or gender of politicians, but their mentality and cultural and political attitude. However, as the film director Holland noted, protesting feminists:

“… mean men, primarily those from the Solidarity generation, proud of their struggle, building democracy, successful transformation, in a word—satisfied with their achievements and satisfied with themselves, unwilling to see that their mistakes, omissions, pride and laziness could have dramatic consequences for many. … Men of this generation have not learned to listen, to listen to voices other than their own, to accept other points of view. The cry of the women has bounced off them as if off a smooth wall. Thinking almost exclusively in terms of political and economic efficiency, they have become unreliable” (Holland, 2020).

According to this characteristic, *dziadersi* are supporters of economic technocratism, activists of the former anti-communist opposition, supporters of more or less overt conservatism and fans of cultivating the myths about the period of neoliberal transformation in Poland in the late 1980s and early 1990s. In this approach, the feminist statements that went beyond this framework not only constituted a threat to a certain social order but could also be perceived as a negation of the dominant liberal-conservative narrative that legitimized and defined the shape of public debate in Poland since the 1990s.

## 7. Discussions: From women's street contestation to social emancipation?

This analysis has shown that the barrier to the implementation of the postulates of the feminist movement in Poland is not only the oppressive right-wing populist government but also the shape of the entire public debate, in which the opposition also reproduces a conservative way of thinking. It has already been pointed out that the opposition was ineffective against the populist government in both Poland and Hungary. It also failed to notice that Polish society was changing faster than the language of the official political debate. The main message of the Women's Strike involved questioning the conservatism of the authorities and the opposition.

What the ruling right and the liberal opposition had in common was that they were both afraid of grassroots social actors and did not want to see their potential or treat them seriously as equal partners in the public debate. In 2020, the Women's Strike became a nationwide event and caused the greatest political crisis in Poland since 2015, when the populist right had taken over power. Thousands of people on the streets (at the peak of the protests on 30 October 2020, over 100,000 people protested on the streets of Warsaw, and around 600,000 in Poland, which was a great success for the organizers given the ongoing COVID-19 pandemic), who questioned not only the logic of the PiS government but also the authority and political influence of the Catholic Church, gave the impression of a revolutionary movement. The scenography of the street protests, where red flags with black lightning (the symbols of the movement) or black flags with red lightning fluttered, resembled the atmosphere of the best traditions of emancipation movements in Europe. The rejection of petty-bourgeois manners and the symbolic questioning of the role of “good girls” by young girls on the streets using slogans such as “Incidentally, I'm pissed off” took this movement far beyond the narrow political dimension. Although the wave of protests in late 2020 and early 2021 did not change the law on the prohibition of abortion in Poland, it fuelled and accelerated cultural and political changes. Muszel and Piotrowski have drawn attention to the long-term cultural impact of the Women's Strike. They have emphasized what consequences grassroots social movements could have on the trajectories in the evolution of social attitudes under the rule of populist authorities (Muszel and Piotrowski, 2022). The numerous Women's Strike demonstrations in the autumn of 2020 may be an illustration of the process in which culture shapes social practices and attitudes, and influences everyday policy in various social areas: public opinion and daily interactions, media coverage and popular culture messages, as well as politics and political actors (Amenta and Polletta, 2019). In social research and analyses of social communication, a lot of attention is paid to how social media and new information technologies shape social attitudes. However, it is worth treating social movements as civil media, which not only publicize matters omitted by state institutions, market and corporate media but also influence messages and topics present in official politics and mainstream media. Mass social movements can also be a network that, contrary to official narratives and systemic obstacles, reaches various sectors of society with messages and gives new meaning and political potential to seemingly common phenomena that were previously devoid of a political context.

Graff and Korolczuk (2022) are right when saying that these changes mainly concern the decreasing authority of the Catholic Church and its importance for the young generation. However, this cultural shift initiated by the 2020 protests proved to be much broader and deeper.

Firstly, society and particularly the young generation became more active and politically involved. The year 2020 turned out to be unique in terms of participation in social protests—nearly a quarter of young people aged 18–24 admitted in a survey carried out in 2021 that they had participated in demonstrations and strikes a year earlier (CBOS, 2021a). It was the highest percentage recorded since the early 1990s and the fall of communism in Poland (Table 1).

**Table 1:** Did you participate in a strike or demonstration in the past year?

	**1993**	**1994**	**1995**	**1996**	**1997**	**1998**	**1999**	**2000**	**2001**	**2002**	**2003**	**2004**	**2005**	**2006**	**2007**	**2008**	**2009**	**2010**	**2011**	**2012**	**2013**	**2014**	**2015**	**2016**	**2017**	**2018**	**2019**	**2020**
Respondents aged of 18–24	0.4	0.0	0.5	4.5	2.1	3.2	0.8	3.8	2.7	1.3	0.0	4.9	4.0	0.0	5.0	2.5	1.4	1.6	1.4	2.0	0.8	3.5	4.6	6.2	6.8	8.5	6.6	23.8
Total respondents	3.4	1.6	0.7	3.0	2.3	1.9	3.6	2.2	1.4	1.2	1.6	2.0	1.9	1.3	2.9	3.4	1.3	1.2	1.7	1.5	2.7	3.1	3.6	5.4	6.3	6.3	6.5	8.3

Affirmative answers in percentage points.

**Figure 5:**
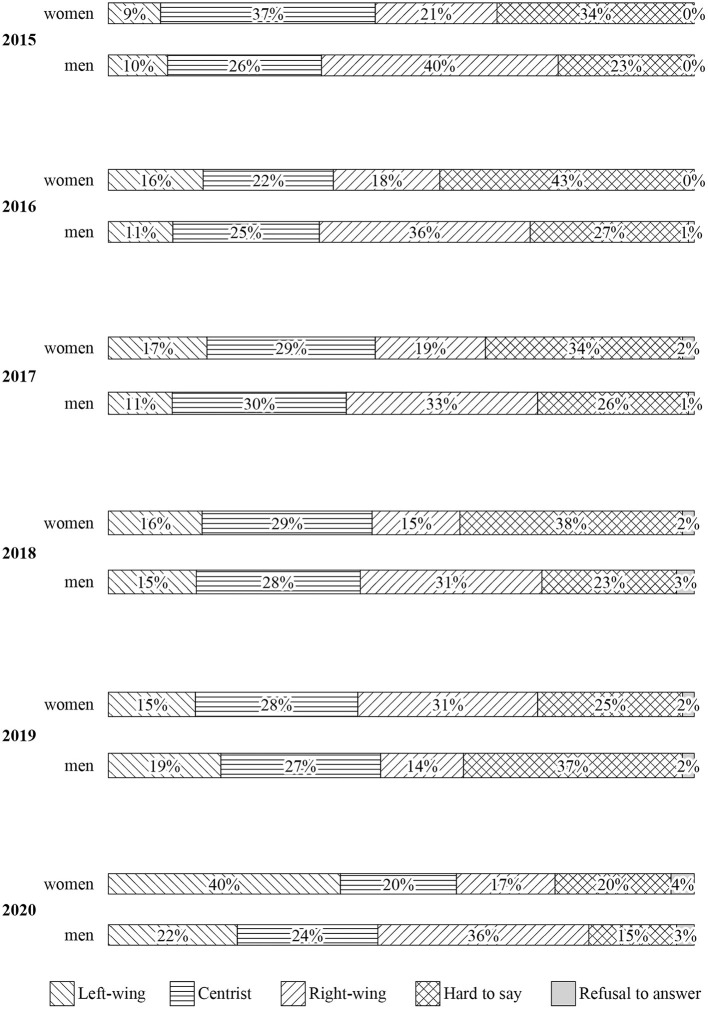
Declared political views of men and women aged 18–24.

Secondly, participation in the protests strengthened the previously observed change in political sympathies among young people in Poland. Since the right-wing populists came to power, the percentage of left-wing sympathies among the young generation of Polish society, particularly among women, has been systematically growing. The breakthrough took place in 2020 when a record 40% of young women admitted that they had left-wing views. This percentage was almost two times lower among men (22%) yet it still increased by over 100% compared to the 2015 data (Figure 5) (CBOS, 2021b).

**Figure 6:**
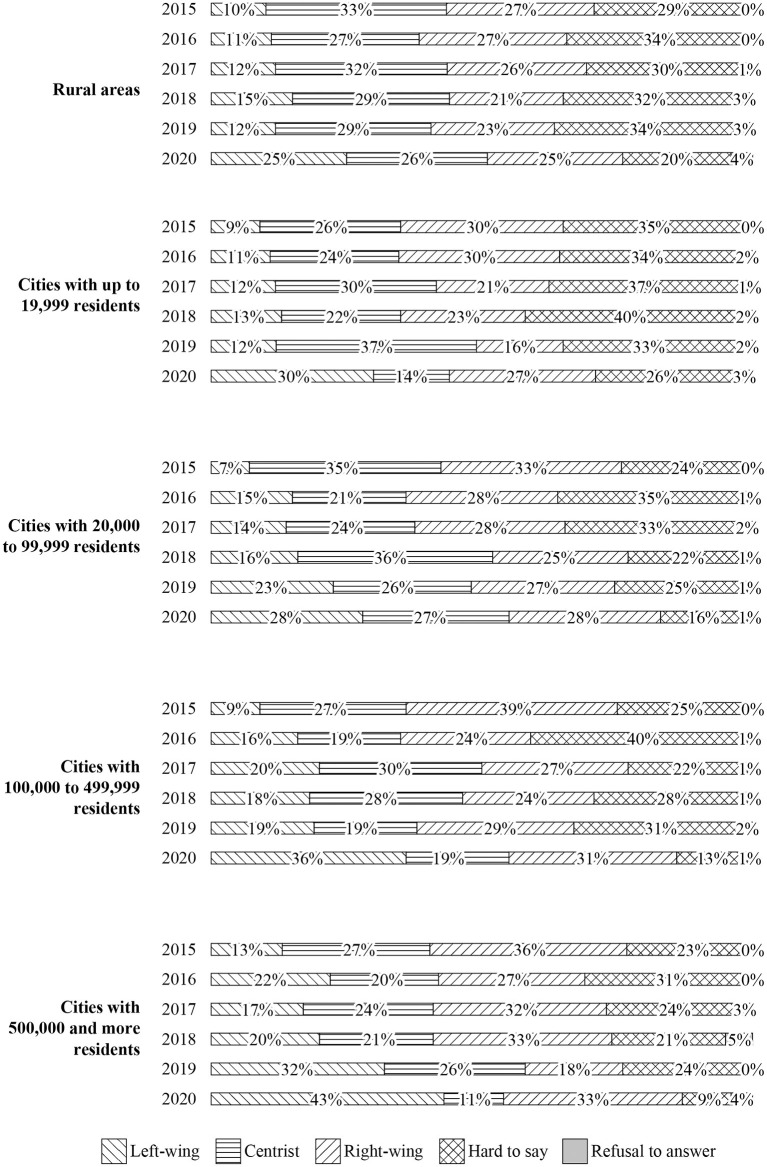
Declared political views of respondents aged 18–24 by place of residence.

The spatial and geographical distribution of these political changes was also interesting. Although the greatest increase in left-wing supporters was in large cities (from 13% in 2015 to 43% in 2020), there was also a systematic increase in support for the left among young people in rural areas (from 10 to 25%) and small towns (from 9 to 30%) (Figure 6) (CBOS, 2021b).

**Figure 7:**
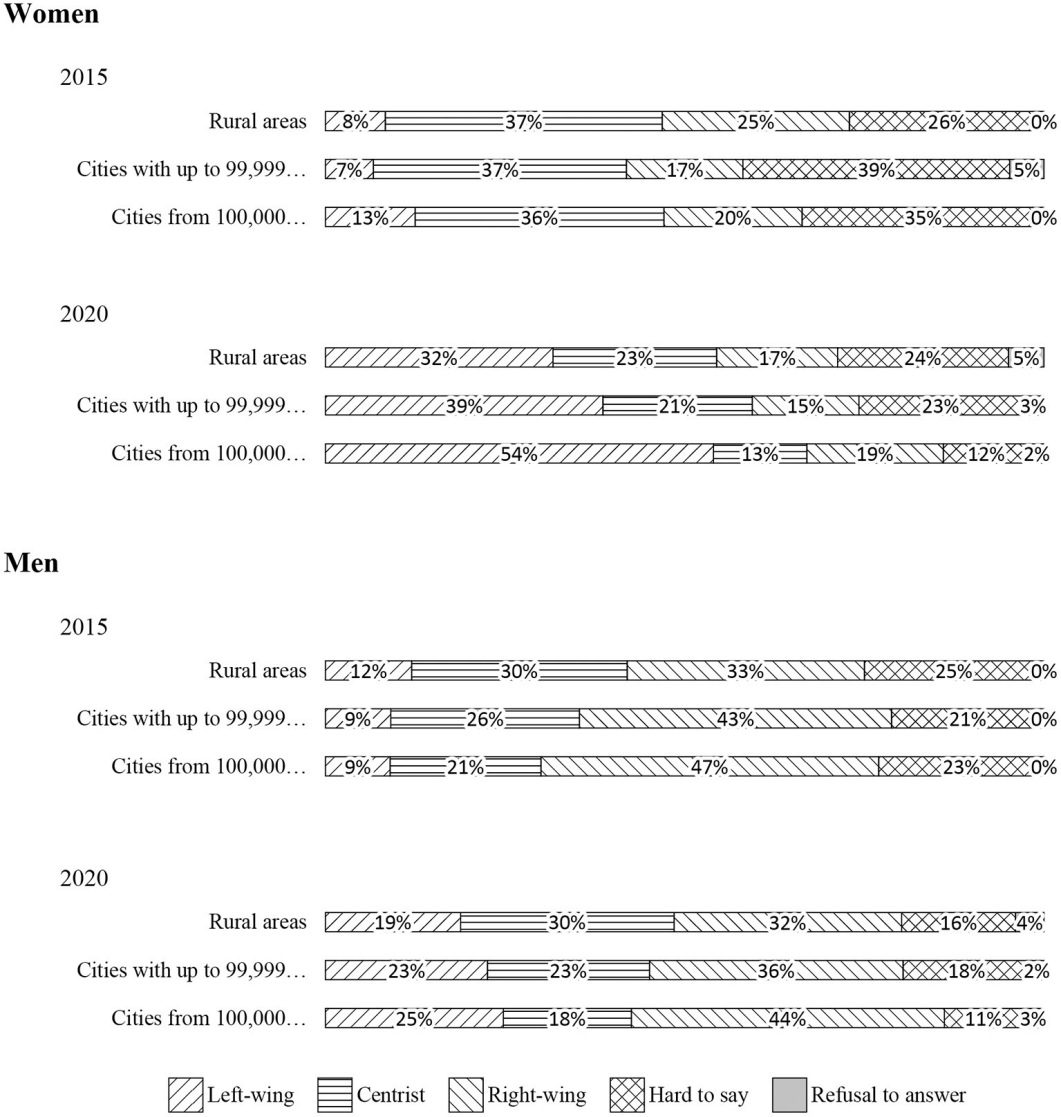
Declared political views of respondents aged 18–24 by gender and place of residence.

The street protests of the Women's Strike also showed the increasing social, cultural and spatial differences between girls and boys. Although the shift to the left took place in all places of residence, gender differences emphasized the increasing divergence between different socio-political worlds of girls and boys. For example, in 2020, in cities with more than 100,000 residents, as many as 54% of girls admitted that they had left-wing sympathies (compared to 25% of young men), and only 19% of girls declared to be right-winged (compared to as many as 44% of young men) (Figure 7). These differences showed the scale of gender and political clash in the young generation.

**Figure 8:**
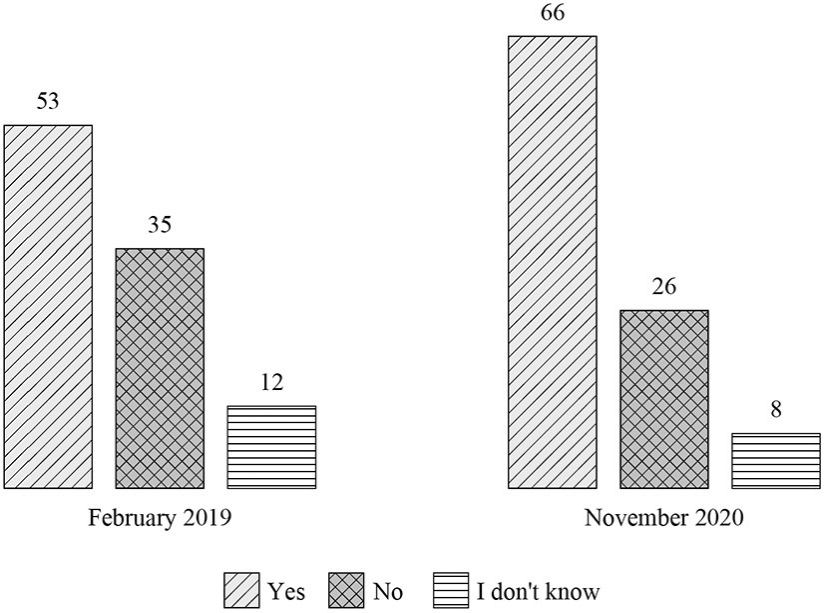
*Should women have the right to terminate a pregnancy up to the 12th week?* Answers in percentage points in Ipsos polls of 14–16 February 2019 and 25–27 November 2020.

**Figure 9:**
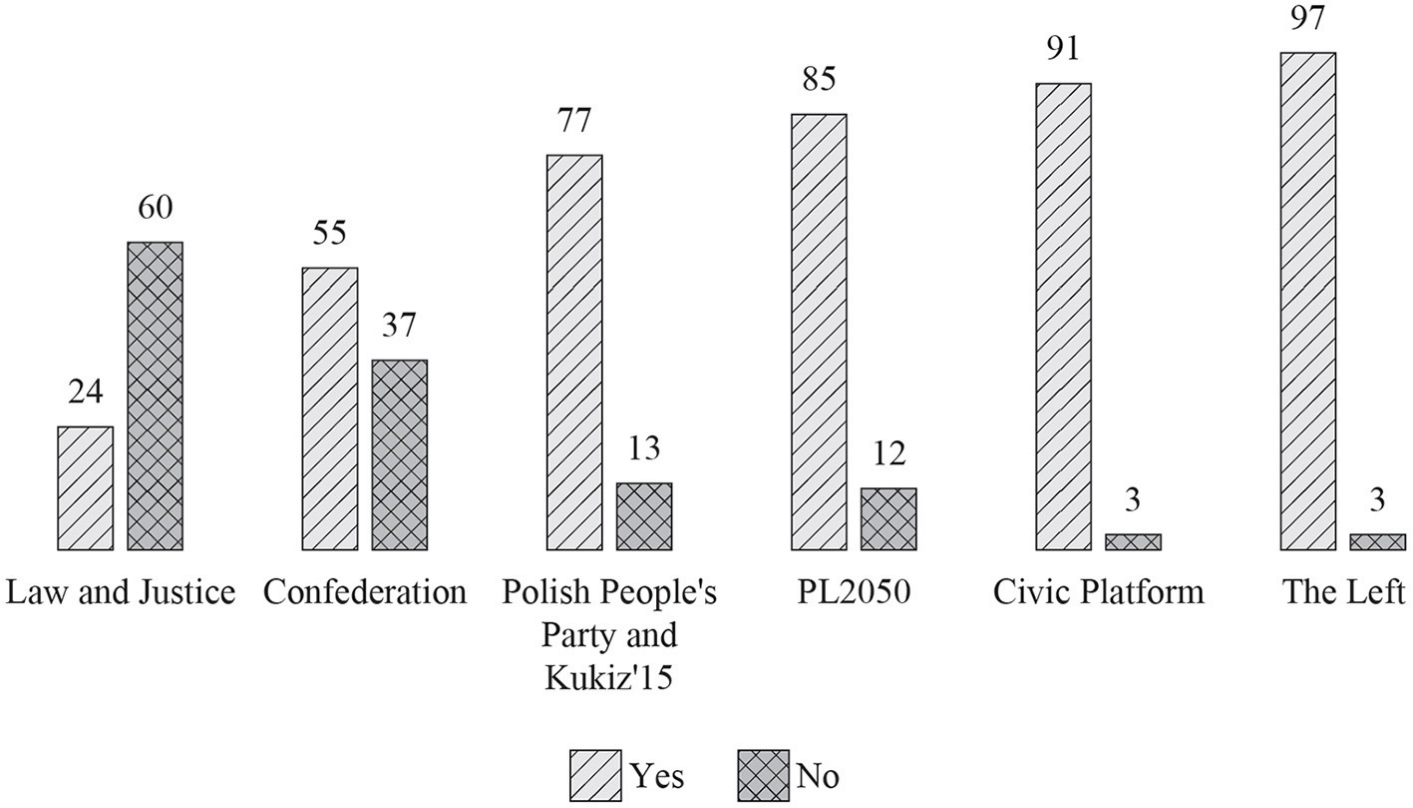
*Should women have the right to terminate a pregnancy up to the 12th week?* Responses of voters of individual parties. Ipsos poll of 25–27 November 2020.

The events of late 2020 also influenced the attitudes of the general Polish society toward the issue of women's right to abortion. In a very short period of time (from spring 2019 to autumn 2020), the percentage of people supporting the right of women to terminate pregnancy up to the 12th week increased significantly (66%) (Figure 8) (Chrzczonowicz, 2020). It turned out that the voters of the opposition parties were more liberal in moral matters than the PiS government and also the opposition parties. In 2020, the number of opponents of the women's right to abortion prevailed only among PiS voters (60%). On the other hand, as many as 97% of left-wing supporters and 91% of voters of the liberal Civic Platform supported the right to abortion under the influence of street demonstrations and the atmosphere that arose in society (Figure 9). These accelerated cultural changes could not go unnoticed by the leaders of the opposition parties. Two years later, Donald Tusk, the leader of the Civic Platform, announced that in the next parliamentary elections to be held in 2023, those who would not support the liberalization of the abortion law would not be placed on the party's electoral list (Rzeczpospolita, 2022). This meant that the liberal opposition completely abandoned the conservative narrative about women's rights.

## 8. Conclusions

The narrative of right-wing populists described in this article using the example of the content of weekly magazines ideologically supporting the PiS party in Poland illustrates the main threads in the message of right-wing populists in various countries: feminists are a threat to the traditional family and social order, the women's movement is another embodiment of neo-Marxism, and the feminist movement (next to the LGBT movement or the ecological movement) is a manifestation of an ideological revolution that is to weaken the national community and tradition. This message may, on the one hand, consolidate conservative and nationalist circles but, on the other, may provoke social protests and further fuel cultural and political conflicts. The example of the Women's Strike in Poland has shown that the pushy propaganda and narrative of right-wing populists can accelerate cultural changes and cause the rejection of conservative models and patterns. This applies not only to the public but also to the opposition parties, which previously presented more conservative and traditional attitudes. Under the influence of the Women's Strike, *dziadersi* underwent a process of cultural modernization.

Considering the short period from 2020 to 2022, it can be said that the Women's Strike led to a steady decline in support for the ruling PiS party by approximately 8–10%. After the events of autumn 2020, PiS no longer gained more than thirty-odd percent, not to mention the level of 40%, which the right-wing populists had previously enjoyed in Poland. Whether the women's issue will help to remove the right-wing populists from power in Poland will be revealed in the autumn of 2023. Certainly, however, the activists of the opposition parties noticed the importance of grassroots protest movements under the conditions of the progressive erosion of democratic principles (Żuk and Pacześniak, 2022). Social movements can transform existing political parties and their programs, establish new political parties and create new hybrid political organizations that have the characteristics of a social movement and parties that contest the entire existing political system (Hutter et al., 2019). How the phenomenon of the Women's Strike will influence the modernization of Polish politics will be shown in the near future. Perhaps the activity of other social movements, such as the environmental movement (Żuk, 2022), attacked in a similar way as the women's movement by the right-wing media in Poland will shape not only the future political agenda but also the form of pursuing future politics as well as the contents and objectives of social policy, family policy, energy policy and environmental policy. All these spheres of public life need to be radically refreshed and cleared of the remnants of both the neoliberal transformation and the post-2015 period of the right-wing populist experiment.

During the communist period, women played a significant role in changing the political system, overthrowing it and questioning the conservatism of the leaders of the former Solidarity movement of the 1980s (Żuk and Żuk, 2018b). Today, the women's movement can contribute to overthrowing not only the rule of the populist right but also the entire conservative order resulting from the neoliberal transformation. This would be a chance for the emancipation of women and also for a fresh breeze of the emancipatory movement in politics combining the postulates of redistribution and recognition.

## Data availability statement

The original contributions presented in the study are included in the article/supplementary material, further inquiries can be directed to the corresponding author.

## Author contributions

PŻ and AP contributed to the conception and design of the study and analyzed press materials and survey data. PŻ collected materials for analysis and prepared the original version of the article based on content analysis. Both authors contributed to the article and approved the submitted version.
